# Update on central factors in myopia development beyond intraocular mechanisms

**DOI:** 10.3389/fneur.2024.1486139

**Published:** 2024-11-18

**Authors:** Rui-Kang Tian, Xiao-Xue Tian, Hai-Bo Yang, Yi-Ping Wu

**Affiliations:** ^1^State Key Laboratory of Ophthalmology, Optometry and Vision Science, Eye Hospital, Wenzhou Medical University, Wenzhou, Zhejiang, China; ^2^School of Clinical Medicine, Shandong Second Medical University, Weifang, China; ^3^Department of Ophthalmology, Nanjing BenQ Hospital, Nanjing, China; ^4^Department of Ophthalmology, Eye Hospital of Shandong First Medical University, Jinan, China

**Keywords:** myopia, intraocular mechanisms, central factors, brain nuclei, refraction

## Abstract

Myopia, a prevalent refractive error, primarily affects children and adolescents, characterized by excessive axial elongation causing distant objects to focus in front of the retina. This review explores the intricate mechanisms beyond intraocular factors, emphasizing the significant role of central factors in myopia development and progression. Intraocular mechanisms involving the retina, RPE/choroid, and sclera are well documented, with these structures playing crucial roles in eye growth regulation. Central factors, including brain structure and function alterations, are increasingly recognized, supported by advanced imaging techniques such as fMRI and rs-fMRI. Clinical findings highlight changes in brain activity and connectivity in high myopia (HM), suggesting neural plasticity or compensatory mechanisms. Animal studies further elucidate central mechanisms, indicating the involvement of specific brain nuclei like the visual cortex and suprachiasmatic nucleus. Understanding these complex interactions between intraocular and central mechanisms is crucial for developing novel therapeutic strategies to inhibit myopia progression and prevent associated complications. This review aims to provide a comprehensive analysis of current research, contributing to a deeper understanding of central factors of myopia.

## Introduction

Myopia is a common refractive error that predominantly affects children and adolescents. It is characterized by excessive axial elongation of the eye, which causes distant objects to focus in front of the retina, resulting in blurred distance vision (1). Myopia is a severe public health concern, as it is a leading cause of visual impairment and blindness. It has a significant impact on the economy and poses a substantial public health burden, particularly in East and Southeast Asia (2–4). Meanwhile HM can lead to additional ocular complications, such as retinal detachment, macular degeneration, fundus pathology, and posterior staphyloma (5). Current treatment options for myopia primarily involve optical correction using glasses or contact lenses. Although interventions such as multifocal contact lenses and atropine can reduce the progression of myopia by up to 60% in some cases, they cannot completely halt its progression (6, 7). Therefore, understanding the mechanisms underlying myopia development is crucial for devising new strategies and therapeutic approaches to inhibit myopia progression and prevent its associated pathological complications.

The development and progression of myopia are influenced by both intraocular mechanisms and central factors. Currently, intraocular mechanisms are widely accepted and primarily involve the retina, retinal pigment epithelium (RPE)/choroid, and sclera. Previous studies have suggested that disruption of the eye-brain connection still induces form-deprivation myopia, indicating that intraocular mechanisms play a major role (8, 9). Meanwhile, an increasing body of evidence suggests that central mechanisms are involved in the regulation of refractive development, yet the specific mechanisms and potential brain regions involved remain unknown. Species specificity leads to different outcomes when applying the same methods to different animal models (10), further complicating the study of central mechanisms in myopia. It is also unclear whether intraocular mechanisms and central factors interact in the development of myopia. This review, while summarizing intraocular mechanisms, provides additional insights into the potential involvement of central factors in myopia.

### Retina

The retina is regarded as a specialized sensory neural tissue capable of detecting optical defocus signals and generating molecular signals specific to the type of defocus (11). This capability allows the retina to determine whether images are accurately focused on it and to locally regulate growth-related optical changes in the eye (12, 13). Applying positive lenses to the eye induces myopic defocus, causing images to focus in front of the retina. This leads to the inhibition of axial elongation and the development of hyperopia. Conversely, negative lenses induce hyperopic defocus when applied to the eye, resulting in axial elongation and myopia (14). The involvement of the retina in the formation and development of myopia encompasses a variety of molecular, cellular, and physiological mechanisms.

#### Dopaminergic mechanisms

Dopamine (DA), a crucial neurotransmitter in the retina, has been implicated in eye growth regulation. Dopamine signaling is influenced by visual input, with multiple retinal circuits contributing to the regulation of dopaminergic amacrine cells and interplexiform cells, which are responsible for DA synthesis and release (15, 16). Retinal dopaminergic neurons receive excitatory input via ectopic synapses formed with ON bipolar cells in the OFF sublamina of the inner plexiform layer (17). Additionally, these neurons are subject to inhibitory regulation through GABAergic and glycinergic amacrine cells (18). There are two potential mechanisms by which DA signaling may interact with visual pathways to modulate eye growth in response to visual stimuli. First, dopamine may bind to DA receptors in specific visual pathways—such as the ON and OFF pathways or rod pathways (19–21), thereby influencing their function and contributing to the development of myopia. Second, visual pathway activity may affect DA release and signaling, thereby influencing myopia progression by altering the dynamics of dopaminergic transmission (22). Dopamine receptors, which are G-protein-coupled receptors, can be divided into two major classes: D1-like and D2-like receptors. D1-like receptors include the D1 and D5 receptor subtypes, while D2-like receptors comprise the D2 and D4 receptor subtypes (23). These receptors are widely expressed in different retinal cell types. Specifically, D1-like receptors are found in bipolar cells, horizontal cells, amacrine cells, and retinal ganglion cells. In contrast, D2-like receptors are located in photoreceptors and retinal pigment epithelium cells. These receptors play crucial roles in modulating visual processing and retinal function. More specifically, D1 receptors are present in type-specific bipolar cells, horizontal cells, a subset of amacrine cells, and retinal ganglion cells. D2 receptors are localized in photoreceptors and dopaminergic amacrine cells. D4 receptors are found in the photoreceptors of mice, while D5 receptors are expressed in the retinal pigment epithelium cells (24). Genetic and pharmacological findings from experiments on mice with myopia indicated that the activation of D1-like and D2-like receptors in specific cell types plays a role in maintaining the balance of the emmetropization process. Excessive activation of D1-like receptors results in hyperopia, whereas excessive activation of D2-like receptors leads to myopia (25–29).

#### Photoreceptor contributions

Both rod and cone photoreceptors are involved in myopia progression. Rods and cones appear to have distinct roles in both refractive development and experimental induced myopia. Cone dysfunction has been linked to increased susceptibility to form-deprivation myopia (FDM) (30). Moreover, the balance between long and middle wavelength-sensitive cones influences refractive development, with disruptions in this balance potentially leading to myopia (31–33). These findings indicated that cone malfunction can contribute to myopic eye growth.

Rod pathways play a crucial role in refractive development and FDM across different light levels in mice. Liang reported that the extension of rod photoreceptor outer segments exerts pressure on the basal lamina of the retinal pigment epithelium, which can lead to thinning of the choroid and occlusion of choroidal vessels, thereby reducing ocular blood flow. This pressure may also extend to the sclera, ultimately contributing to the progression of myopia (34). Meanwhile, inducing form deprivation in the peripheral region of the monkey eye, which is primarily composed of rods, produced a level of myopia similar to that seen when the entire visual field was subjected to deprivation (35). Warwick used patch clamp technology found that under normal conditions, the cone inputs showed strong surround inhibition, while the rod pathway inputs lacked surround responses. However, during D1 receptor blockade, surround activation was observed to arise from the primary rod pathway (36). Anthocyanins promote the regeneration of rhodopsin in the outer segments of frog rod cells. Furthermore, in a chick model of myopia induced by negative lenses, anthocyanins have been shown to inhibit the elongation of axial length (37). Some studies also propose that dopamine release is primarily regulated by rod photoreceptors, with rods inhibiting DA release under low light and stimulating it in very bright conditions (38). Their findings highlight the high-threshold nature of light-induced DA release, which is significantly influenced by rod function. It was also found that rod dysfunction like Gnat1−/− mice did not develop myopia upon form deprivation (FD) (39). Overall, these findings indicate a possible link between rod cells, the rod pathway, and the development of myopia.

#### Müller glia and myopia

Müller glia, the principal macroglial cells in the retina, have emerged as significant players in ocular growth regulation (40). Müller cells provide radial structural support (41). An important vessel supplying the outer retina-choroid-sclera is the short posterior ciliary artery. When the retina’s oxygen demand increases, the short posterior ciliary artery delivers more oxygen to the outer retina, consequently reducing oxygen supply to the sclera. This triggers the release of factors by retinal glial cells, which act on the outer ocular layers. These factors, in conjunction with scleral factors, contribute to the process of scleral remodeling. Müller cells were observed to secrete different factors based on the degree of hypoxia (42). Müller cells produced a protein factor that downregulated pigment epithelium derived factor in mild hypoxia, while pigment epithelium derived factor exhibited neurotrophic under severe or chronic hypoxia (43).

#### Intrinsically photosensitive RGCs

Research indicates that ipRGCs integrate signals from melanopsin as well as rods and cones, influencing both axial length (AL) via melanopsin signals and corneal radius of curvature (CRC) via rod/cone-driven signals. Ablation of certain cell subtypes demonstrated that M1 subtype cells, and maybe M2/M3 subtype cells, play a role in ocular development. IpRGCs are also known to regulate “local” intraretinal rhythms, as demonstrated by the reduction in daily fluctuations in cone ERG and the disturbance of clock gene rhythms in mice lack melanopsin (44, 45). Meanwhile, ipRGCs have the ability to transmit light signals to the outer retina by interacting with upstream dopaminergic amacrine cell (DAC) (46). Chakraborty discovered that FD mice with melanopsin deficiency had decreased levels of retinal DA and DOPAC. However, the absence of melanopsin alone did not alter retinal DA levels. Hence, an intact ipRGC-DAC interplay is crucial for refractive development in mice.

#### Retinal ganglion cells

Currently, more than 40 types of RGCs have been identified in the mouse retina (47). These cells are responsible for detecting, encoding visual information and transmitting it to the brain. Norton has shown that despite blocking RGC action potentials with tetrodotoxin (TTX), FDM still occurs (8). However, researchers recently have discovered that RGCs may also play a role in local retinal mechanisms. Using patch-clamp techniques and different defocus stimuli, Pan discovered that defocused image decreased probability of spikes in ON, OFF, and ON–OFF RGCs, this change in signaling may be an initial step in myopia development (48). Pan focused on αRGCs because they are known to play a critical role in visual processing. αRGCs can encode differences between focused and defocused images in both normal and myopic retinas (49), and they possess the largest somata, broader dendritic fields, and a uniformly distributed mosaic pattern across the retina (50, 51). The axial elongation of the myopic retina can significantly affect the properties of αRGCs, which are considered to reflect the characteristics of all retinal ganglion cells. OPN5-expressing RGCs were also found to be crucial for emmetropization in mice. The study found that the protective impact of violet light on myopia is contingent upon the presence of OPN5 (52). They found that violet light exposure mitigates the reduction in choroidal thickness caused by hyperopic defocus, and this effect is also dependent on retinal OPN5. The violet light/OPN5 pathway may play a protective role in preventing excessive ocular responses to defocus by regulating choroidal thickness. Another study by Adam demonstrated that ON-delayed retinal ganglion cells exhibit high sensitivity to high spatial frequency patterns presented over a large receptive field, suggesting that these cells may function as defocus detectors in the mouse retina (53).

## RPE/choroid

The RPE synthesizes and releases multiple growth factors and cytokines that have been linked to the regulation of growth, such as IGF-1, TGF-*β*, FGF, VEGF, and bone morphogenetic proteins (54). Notably, bone morphogenetic proteins family members exhibit a rapid bidirectional reaction to contrasting growth signals, and their expression can be modulated by DA, suggesting a potential route through which the RPE might transmit growth signals from the retina (55). Meanwhile, dynamic changes in fluid movement within the RPE are noted during phases of altered growth. Liang discovered that during recovery from FDM, there is heightened fluid accumulation and swelling within the retina, RPE, and choroid, accompanied by ultrastructural reorganization of the RPE basal lamina. This suggests dynamic alterations in fluid transport across the RPE, which acts as a barrier, permitting controlled exchange of ions and water between the subretinal space and the choroid by adjusting its ionic channels (56).

The highly vascular choroid, sandwiched between the retina and the sclera, supplies oxygen and nutrients to the RPE, the outer retina, and the sclera (57). The choroid influences the length of the eye by altering its own thickness. This adjustment shifts the retina either forward or backward, thereby aligning the photoreceptors to the correct focal plane. Zhou’s study found that increased choroidal blood perfusion can mitigate scleral hypoxia, thereby inhibiting the progression of myopia (58). Conversely, a reduction in choroidal blood perfusion may lead to scleral hypoxia, which in turn can induce the transdifferentiation of scleral fibroblasts into myofibroblasts (59).

### Sclera

The sclera is a highly resilient and structurally complex connective tissue whose primary function is to provide a firm and stable environment for the retina. When there are defocus factors or other visual stimuli such as FD, light intensity, contrast sensitivity, and other factors, they trigger a retinal cascade response that ultimately transmits to the sclera (60, 61). This leads to the expression of related scleral genes, such as HIF-1α, MMPs, TIMPs, and TGF-*β*, resulting in corresponding changes in scleral structure, biomechanics, and composition, a process known as scleral remodeling. Wu discovered that signaling of HIF-1α promotes myopia by causing fibroblasts to transform into myofibroblasts, and that treatment targeting hypoxia prevented the molecular changes associated with HIF-1α, thus stopping the progression of myopia (62).

The intraocular mechanisms are also reflected in the localized response within the eye. Even when the optic nerve is transected or the RGC action potential is chemically blocked, preventing signal generation and transmission to the brain, FDM still occurs (8, 63). Furthermore, if diffusers or negative lenses are applied to cover only half of the retina, only the covered half of the eye becomes enlarged and develops myopia (64), and if positive lenses cover only half of the retina, only that covered half exhibits inhibited eye growth.

Taken together, the intraocular mechanisms of eye growth regulation are primarily mediated through a retinal signaling cascade, which subsequently influences the RPE/choroid and ultimately the sclera, acting as an effector to regulates eye growth through scleral remodeling (65).

### The role of central factors in myopia: clinical findings

With the advancement of imaging technologies, our understanding of myopia has been revolutionized, allowing for a detailed investigation of the brain’s involvement in myopia. Table 1 shows the results of the clinical findings between myopia and brain regions. Functional magnetic resonance imaging (fMRI), resting-state fMRI (rs-fMRI), and amplitude of low-frequency fluctuations (ALFF) have been pivotal in elucidating the central neural mechanisms associated with myopia. These advanced imaging techniques provide comprehensive insights into how myopia impacts brain structure and function, thereby enhancing our understanding of its underlying pathophysiology.

**Table 1:** Summary of clinical findings on the association between myopia and brain regions.

Research titles	Year	Author	Assessment method	Involving brain regions	Participants
Altered spontaneous brain activity pattern in patients with high myopia using amplitude of low-frequency fluctuation: a resting-state fMRI study	2016	Huang et al.	ALFF method with rs-fMRI	Right inferior and middle temporal gyrus, left middle temporal gyrus, left inferior frontal gyrus/putamen, right inferior frontal gyrus/putamen/insula, right middle frontal gyrus, right inferior parietal lobule, bilateral midcingulate cortex, left postcentral gyrus, left precuneus/inferior parietal lobule	High Myopia
Abnormal resting-state functional network centrality in patients with high myopia: evidence from a voxel-wise degree centrality analysis	2018	Hu et al.	Voxel-wise degree centrality method with rs-fMRI	Right inferior frontal gyrus/insula, right middle frontal gyrus, right supramarginal/inferior parietal lobule, right cerebellum posterior lobe, left precentral gyrus/postcentral gyrus, right middle cingulate gyrus	High Myopia
Altered amplitude of low-frequency fluctuations and default mode network connectivity in high myopia: a resting-state fMRI study	2020	Zhang et al.	ALFF method with rs-fMRI	Left inferior temporal gyrus, bilateral rectus gyrus, bilateral middle temporal gyrus, left superior temporal gyrus, left angular gyrus	High Myopia
Exploration of abnormal dynamic spontaneous brain activity in patients with high myopia via dynamic regional homogeneity analysis	2022	Ji et al.	Dynamic regional homogeneity analysis with rs-fMRI	Left fusiform gyrus, right inferior temporal gyrus, right Rolandic operculum, right postcentral gyrus, right precentral gyrus	High Myopia
Altered time-varying local spontaneous brain activity pattern in patients with high myopia: a dynamic amplitude of low-frequency fluctuations study	2023	Zhang et al.	dALFF approach with rs-fMRI	Left inferior frontal gyrus (orbital part), left lingual gyrus, right anterior cingulate and paracingulate gyri, right calcarine fissure and surrounding cortex, left thalamus, left paracentral lobule, left inferior parietal lobule (except supramarginal and angular gyri)	High Myopia
Altered functional connectivity density in high myopia	2016	Zhai et al.	Functional connectivity density mapping and seed-based correlation analysis with rs-fMRI	Posterior cingulate cortex/precuneus, inferior temporal gyrus, supramarginal gyrus, rostrolateral prefrontal cortex	High Myopia
Abnormal Large-Scale Neuronal Network in High Myopia	2022	Ji et al.	rs-fMRI	Visual network, dorsal attention network, auditory network, sensorimotor network, default mode network, salience network, executive control network, cerebellar network	High Myopia
Effect of lens-induced myopia on visual cortex activity: a functional MR imaging study	2011	Mirzajani et al.	fMRI	Occipital visual cortex	Emmetropic volunteers
Effect of induced high myopia on functional MRI signal changes	2017	Mirzajani et al.	fMRI	Occipital visual cortex	Emmetropic volunteers
Voxel-based analysis of regional gray and white matter concentration in high myopia	2012	Li et al.	Voxel-based morphometry with high-resolution anatomic MRI	Left calcarine area, right parietal lobe, right prefrontal area	High Myopia
Evidence of cortical thickness reduction and disconnection in high myopia	2020	Wu et al.	High-resolution T1 and rs-fMRI	Left middle occipital gyrus, left inferior parietal lobule, right inferior temporal gyrus, right precuneus, right primary visual area 1, right superior temporal gyrus, right superior parietal lobule, right occipital pole, right the primary motor cortex, parietal operculum	High Myopia
Brain activation of eye movements in subjects with refractive error	2010	Nelles et al.	fMRI	Bilateral frontal and parietal eye fields, supplementary eye fields, bilateral extrastriate cortex	Subjects with refractive error
Comparison of intrinsic brain activity in individuals with low/moderate myopia versus high myopia revealed by the amplitude of low-frequency fluctuations	2020	Cheng et al.	ALFF method with rs-fMRI	Bilateral rectal gyrus, right cerebellum anterior lobe/calcarine, bilateral thalamus, left white matter (optic radiation), right prefrontal cortex, left primary motor cortex /primary somatosensory cortex, bilateral parahippocampal gyrus, bilateral posterior cingulate cortex, bilateral middle cingulate cortex, bilateral frontal parietal cortex	Low/moderate myopia patients and high myopia patients
Brain Activation Induced by Myopic and Hyperopic Defocus From Spectacles	2021	Kang et al.	fMRI	Right precentral gyrus, right superior temporal gyrus, left inferior parietal lobule, and left middle temporal gyrus	Ametropic group

#### fMRI

The fMRI boasts spatial resolution at the millimeter level and temporal resolution at the second-order level. By detecting fluctuations in MRI signals caused by changes in local blood oxygenation, blood flow, and volume due to neuronal activity in the brain, it allows for the non-invasive measurement of the location, intensity, and dynamic changes of various functional activities (66).

#### Rs-fMRI

Rs-fMRI is a non-invasive neuroimaging technique that measures brain activity by detecting changes in blood flow during rest and activity. This method can accurately locate cortical areas of brain activity and track signal changes in real time. Widely used to explore cognitive characteristics (67), rs-fMRI offers the benefits of direct signal collection and the ability to identify functional areas across diverse patient groups, making it advantageous over other fMRI methods (68).

#### Amplitude of low-frequency fluctuations and dALFF

ALFF, based on detecting spontaneous neuronal activity through blood oxygen level-dependent signals, is a reliable indicator of local brain activity (69). It detects brain function within the 0.01–0.08 Hz frequency range and is widely used to evaluate neuropsychiatric and ophthalmic diseases, such as optic neuritis (70), glaucoma (71), and comitant strabismus (72). The dynamic ALFF (dALFF) technique, combining ALFF with the “sliding-window” method, innovatively illustrates time-varying local brain activity (73), which can indicate neural activity intensity and excitability in specific cerebral cortex regions (74).

## Altered brain activity and connectivity in high myopia

Recent studies have revealed notable disparities in spontaneous brain activity between individuals with HM and those without, providing insights into the neurobiological foundations of myopia.

Huang et al. discovered that individuals with HM exhibited significantly reduced ALFF in regions such as the right inferior and middle temporal gyrus, left middle temporal gyrus, left inferior frontal gyrus/putamen, right inferior frontal gyrus/putamen/insula, right middle frontal gyrus, and right inferior parietal lobule. Conversely, higher ALFF was observed in the bilateral midcingulate cortex, left postcentral gyrus, and left precuneus/inferior parietal lobule (75). In addition, Zhai’s study found that HM patients exhibited reduced functional connectivity density in the posterior cingulate cortex/precuneus, inferior temporal gyrus, supramarginal gyrus, and rostrolateral prefrontal cortex while decreased functional connectivity was found between the supramarginal gyrus and rostrolateral prefrontal cortex, and also between the ventral attention network and the frontoparietal control network (76). In a similar vein, Hu et al. used the voxel-wise degree centrality (DC) method and found that individuals with HM exhibited significantly lower DC values in the right inferior frontal gyrus/insula, right middle frontal gyrus, and right supramarginal/inferior parietal lobule. On the other hand, higher DC values were noted in the right cerebellum posterior lobe, left precentral/postcentral gyrus, and right middle cingulate gyrus (77). These findings suggest that HM is associated with altered activity in brain regions involved in language processing and attentional control. This may imply that HM patients experience neural adaptations in these areas.

By using rs-fMRI, Zhang et al. reported significantly increased ALFF in the left inferior temporal gyrus, bilateral rectus gyrus, bilateral middle temporal gyrus, left superior temporal gyrus, and left angular gyrus in HM patients compared to controls (67). Ji’s research using rs-fMRI demonstrated that individuals with HM exhibited neural activity dysfunction both within and between distinct brain networks, especially the default mode network and cerebellum (78). Ji et al. also analyzed HM patients using dynamic regional homogeneity (dReHo) and found significantly greater dReHo values in the left fusiform gyrus, right inferior temporal gyrus, right Rolandic operculum, right postcentral gyrus, and right precentral gyrus compared to healthy controls (68). Another study by Zhang using the dALFF approach revealed decreased dALFF variability in the left inferior frontal gyrus (orbital part), left lingual gyrus, right anterior cingulate and paracingulate gyri, and right calcarine fissure and surrounding cortex in HM patients. Conversely, increased variability was found in the left thalamus, left paracentral lobule, and left inferior parietal lobule (73). Therefore, individuals with HM may experience impairments in visual, cognitive, attentional control, and motor balancing abilities. This evidence supports the role of brain networks in the pathophysiological mechanisms of HM.

In summary, these studies collectively indicate that HM is associated with widespread abnormalities in spontaneous brain activity across various regions. These changes likely reflect the underlying neurobiological alterations that affect language comprehension, cognitive functions, attentional control, visual and motor balance functions in HM patients (79–85).

### Impact of high myopia on brain structure

Li et al. conducted a voxel-based analysis to compare regional gray matter and white matter concentrations in HM patients versus controls (86). The study found an increased concentration of white matter in HM patients, primarily in the calcarine area, with smaller increases in the prefrontal and parietal lobes. Wu’s study provided evidence of cortical thickness reduction and disconnection in visual centers and processing areas in HM patients. Additionally, an increase in cortical thickness was observed in the left multimodal integration region (87).

### Impact of induced myopia on visual cortex activity

Mirzajani et al. demonstrated that blur induced by myopia significantly impacts fMRI experimental outcomes. Their study explored how mild and moderate lens-induced defocus affected the activity of the occipital visual cortex when exposed to a visual stimulus with moderate spatial frequency. They found that even a slight induced myopia of +1D notably influences visual cortex activity (88). Building on this, Mirzajani examined the effects of lens-induced high myopia on occipital visual cortex activity with two different spatial frequency visual stimuli. For a stimulus with 1.84 cycles per degree, lens-induced high myopia significantly reduced visual cortex activity (*p* = 0.01). However, for a 0.34 cycles per degree stimulus, there was no significant effect [*p* = 0.17; (89)]. Overall, these studies emphasize the significant effect of myopia on visual cortex activity.

Nelles et al. examined how refractive error affects cortical regulation of an oculomotor task using fMRI. The results showed increased activation in the bilateral frontal and parietal eye fields, supplementary eye fields, and bilateral extrastriate cortex among individuals with refractive errors compared to those with normal vision (90). Cheng et al. compared intrinsic brain activity in individuals with low/moderate myopia versus HM using the ALFF method (91). The findings indicated that individuals with mild to moderate myopia and HM had abnormal intrinsic brain activities in regions associated with the limbic system, default mode network, and thalamo-occipital pathway. Kang et al. assessed neural changes induced by myopic and hyperopic defocus stimuli. They found that myopic defocus significantly increased regional cerebral blood flow in the right precentral gyrus, right superior temporal gyrus, left inferior parietal lobule, and left middle temporal gyrus (66), indicating enhanced blood perfusion in visual attention-related regions.

Clinical evidence strongly supports the association between myopia and central neural changes. Consistent findings across different imaging modalities suggest that myopia involves both functional and structural alterations in the brain. These changes may reflect compensatory mechanisms, neural plasticity, or disruptions in normal brain function due to altered visual input.

### Central factors in myopia: findings from animal studies

Animal experiments that sever the connection between the retina and brain reveal that the regulation of visually-driven eye growth and emmetropization is primarily governed by intraocular mechanisms. For instance, Raviola and Wiesel demonstrated that lid-fusion myopia developed in *Macaca mulatta* following optic nerve section (ONS) surgery (92). Subsequent studies using the chick model revealed that performing ONS surgery did not inhibit the progression of FDM or lens-induced myopia (93, 94), suggesting that central communication is not essential for the development of myopia. However, there is also evidence suggesting that central mechanisms play a regulatory role in emmetropization. Gong’s research using optic nerve crush (ONC) to block visual input in mice found that recalibrating the refractive set-point in both directions led to significant refractive changes in the majority of animals. Specifically, 54.5% developed significant myopia (<−3 D) and 18.2% exhibited significant hyperopia (> + 3 D), primarily due to changes in ocular AL (10). McFadden discovered that the gain control in response to FD was significantly altered by ONS. Compared to the sham group, ONS markedly enhanced the response to FD. These findings suggest that advanced visual centers are probably involved in the fine-tuning of eye growth and thereby influence refractive status (9). Dillingham conducted experiments on chicks using electrolytic lesions to target the IOTr nucleus, a limited subset of ectopic neurons analogous to the neurons of the mammalian superior colliculus (SC) (95). The findings revealed that chicks with the highest percentage of successful lesions showed notable axial hyperopia in the treated eye relative to the control eye.

In addition to physical methods, chemical approaches also support this hypothesis. For instance, the injection of TTX or colchicine, which blocks the generation of RGC action potentials, and thereby prevents the transmission of information to higher visual centers, has been shown to have similar effects (96, 97). Norton’s research on tree shrews demonstrated that intravitreal injection of TTX resulted in a significant shift toward hyperopia (8). In normal visual environments, intravitreal injection of colchicine in chicks led to axial elongation and the development of myopia (97, 98). Liu’s research on developing mice demonstrated that selective ablation and activation of ipRGCs induced opposite refractive shifts, leading to myopia and hyperopia. These changes were achieved by modulating the CRC and AL in mice. These experiments collectively suggest that changes in the integrity of eye-brain neural connections can affect refractive status. However, the broad and non-specific nature of methods prevents the identification of specific brain nuclei involved in this process. The close proximity of the ophthalmic artery to the optic nerve raises concerns about potential impacts on blood supply (99). Meanwhile, ONS surgery not only disrupts RGCs efferents to the primary visual centers of the brain but also midbrain centrifugal visual system axons terminating in the retina. Chemical methods, such as using TTX or colchicine to block action potentials, also affect ON and OFF cone bipolar cells, amacrine cells, and ganglion cells due to their influence on sodium ion channels (100). Moreover, the need for continuous daily intravitreal injections of TTX can exert mechanical pressure on the eye. Table 2 summarizes the results of animal models with interventions and refraction changes.

**Table 2:** Summary of experimental interventions and refractive state changes in animal models of myopia.

Author	Year	Intervention Method	Species	Treatment Effect	Reference
Raviola et al.	1985	ONS with lid-fusion myopia	*Macaca mulatta*	Developing toward myopia	92
Troilo et al.	1987	ONS only	Chicks	Developing toward severe hyperopia	63
Wildsoet	2003	ONS only	Chicks	Developing toward hyperopia and reduced VCD	94
		ONS with FD	Chicks	Exaggerated AL changes	94
Choh et al.	2006	ONS with LIM	Chicks	Exaggerated AL changes	93
Norton et al.	1994	TTX only	Tree shrews	Reduced AL changes	8
		TTX with MD	Tree shrews	Developing toward myopia	8
Fischer et al.	1999	Colchicine only	Chicks	Developing toward myopia and axial elongation	98
		Colchicine with FD	Chicks	Reduced AL compensation	98
Choh et al.	2008	Colchicine only	Chicks	Exaggerated AL changes	97
		Colchicine with LIM	Chicks	Reduced AL compensation	97
Gong et al.	2020	ONC only	Mouse	Re-calibrated the refraction in a bidirectional manner	10
McFadden et al.	2020	ONS with FD	Guinea pig	Increased response to FD	9
Dillingham et al.	2013	Electrolytic nucleus lesions	Chicks	Developing toward axial hyperopia	95
Liu et al.	2022	Selective ablation and activation of ipRGCs	Mouse	Developing toward myopic and hyperopic by modulating CRC and AL	45
Jiang et al.	2021	Genetic manipulation	Mouse	VL suppresses LIM via OPN5	52

VCD, vitreous chamber depth; TTX, tetrodotoxin; MD, monocular deprivation; AL, axial length; VL, violet light; CRC, corneal radius of curvature; LIM, lens induced myopia; ONS, optic nerve section.

### Potential central mechanisms involved in myopia development

In avian species, centrifugal visual fibers originating from the brain and projecting to retinal neurons are involved in early eye growth. Although these fibers are very limited in number in mammals (101), they exhibit extensive branching within the retina, encompassing almost the entire retinal region (102). Research has confirmed that these centrifugal visual fibers are dopaminergic and can interact with DACs (103), thereby regulating eye growth by modulating retinal DA release levels. Additionally, central mechanisms may influence eye growth through melanopsin-expressing ipRGCs, which project to the suprachiasmatic nucleus (SCN) and are involved in light-induced circadian rhythms. Current literature indicates that normal refractive development relies on specific daily rhythms of eye growth, and disruption of these rhythms can lead to excessive axial elongation. Meanwhile, the involvement of ipRGCs on ocular growth may be linked to their extensive interaction with retinal blood vessels. Recent studies have shown that some ipRGC processes are near the intermediate capillary plexus and that melanopsin mediates light-induced relaxation in blood vessels (104, 105). Thirdly, degeneration of RGCs could impair the retina’s ability to detect optical defocus and modify the signals generated.

### Potential brain nuclei involved in myopia development

Visual information received by the eyes is transmitted through the visual signal pathways. The brain processes visual information in parallel at three distinct levels: individual neurons, cell types, and neural pathways. Information processing at the cell type level begins in the retina. Visual signals travel via the optic nerve, formed by the axons of RGCs. After partially crossing at the optic chiasm, these fibers form the optic tract, which then transmits the signals to various brain nuclei such as the lateral geniculate nucleus (LGN), SC, SCN, and visual cortex.

The SC is a conserved visual center within the mammalian visual system. It not only receives projections from RGCs but also integrates input from the visual cortex, serving as a crucial hub for information processing and integration (106, 107). Approximately 90% of ganglion cells project directly to the SC (108), which is organized into alternating layers of fibers and cell bodies. The superficial layers receive visual signals, while the intermediate and deep layers process inputs from primary motor, somatosensory, and auditory cortices. The SC mediates various physiological functions, including eye movements, innate fear responses, and sleep regulation (109, 110). In most vertebrates, the extensive inputs and outputs of the SC suggest that the SC can influence almost the entire neural axis. Lee investigated how induced blur affects the response efficiency of midbrain cells. While a small subset of the cells showed minimal changes in their response patterns when subjected to either hyperopic or myopic blur, approximately 86% experienced a significant decline in response efficiency (111). Gehr’s research demonstrated that both excitatory and inhibitory neurons in the superior colliculus receive similarly robust inputs from retinal ganglion cells, and that the same wiring principles govern RGC innervation of both types of SC neurons (112). Li′s study indicated that the superficial layers of the superior colliculus respond to various visual stimuli, such as blue and green flashes (113). Additionally, previous research suggested that flickering blue light may play a role in the progression of myopia (60). Therefore, it is conceivable that the SC may play a role in the development of myopia.

The LGN serves as a critical relay station for visual signals entering the visual cortex. It is responsible for further processing of visual information, where the signals are analyzed, encoded, and then transmitted to the primary visual cortex via the optic radiations. The mammalian retina transmits the majority of visual stimulus information to two brain regions: the dorsal lateral geniculate nucleus (dLGN) and the SC. Studies have shown that SC neurons can acquire most of the information sent by the retina to the dLGN, but not vice versa. Animal studies have demonstrated a reduction in the size of ocular dominance columns within the primary visual cortex, as well as cellular atrophy in the layers of the LGN corresponding to the deprived eye (114).

The SCN is closely linked to circadian rhythms and has a well-established connection to visual functions. Previous studies have demonstrated that disruptions in the eye’s daily growth rhythms can lead to excessive axial elongation (115). It has also been reported that the amplitude of diurnal variations in the choroid is correlated with axial length in adults with myopia. This suggests that circadian rhythm disruption may be involved in the pathogenesis of myopia (116). Previous research has been documented that M1 type ipRGCs primarily project to non-image forming visual regions of the brain, including the SCN (117). Importantly, M2 type ipRGCs were observed to project to both non-image forming visual regions, such as the SCN, as well as image-forming areas like the dLGN and SC (118). Additionally, Liu’s research revealed that cell type–specific ablation studies demonstrated that M1 subtype cells, and potentially M2/M3 subtype cells, play a role in ocular development (45). Li proposed a hypothesis that ocular rhythms are regulated locally and indirectly through the SCN, which receives input from ipRGCs (119). In mammals, the central circadian pacemaker is located in the SCN (120). It has been reported that the SCN, upon receiving light and time-of-day information from ipRGCs, initiates the central clock. This provides an anatomical basis and a potential mechanism for the involvement of the ipRGC-SCN circuit in neuronal regulation.

**Figure 1:**
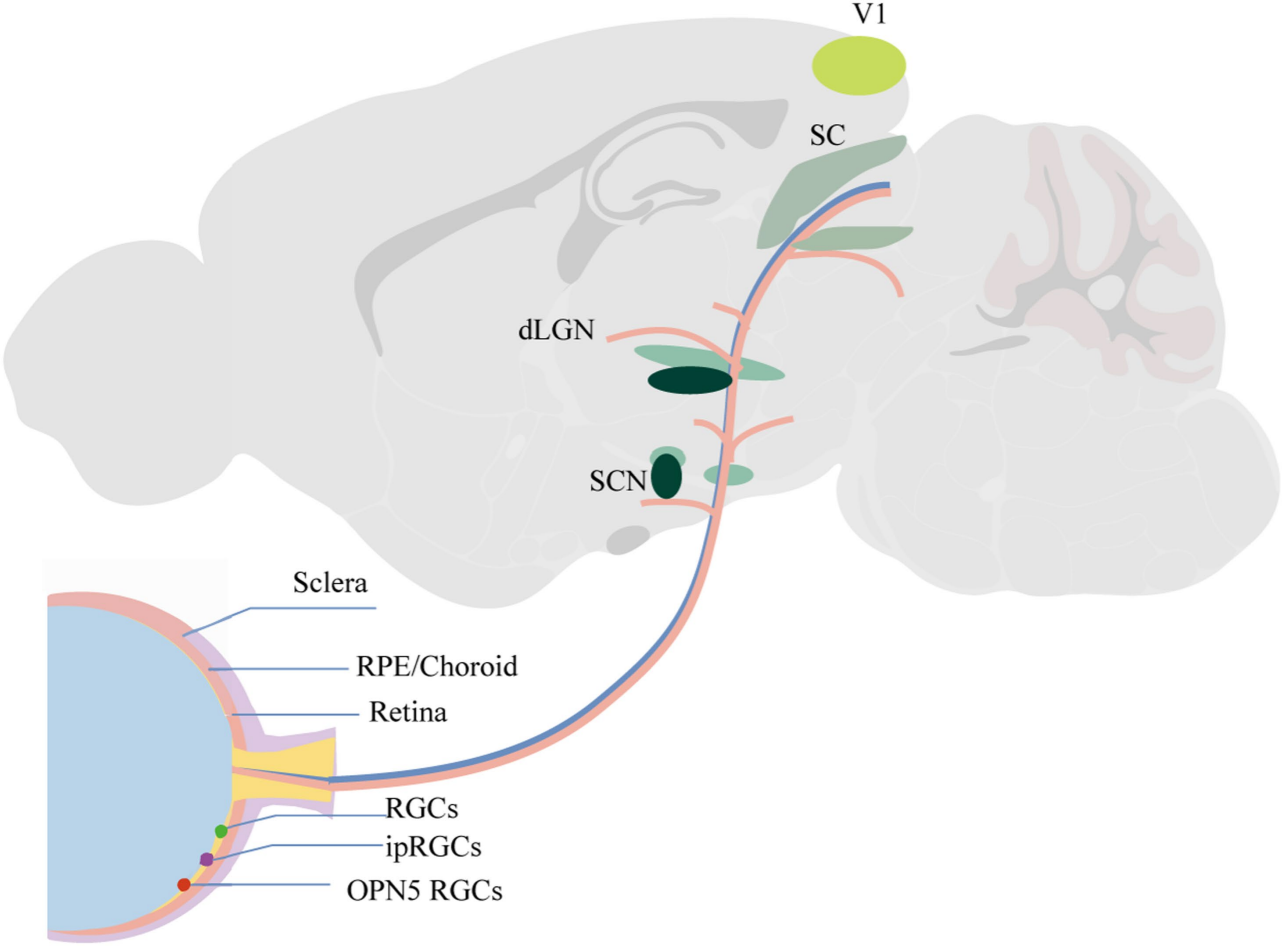
Intraocular structures and potential central pathways involved in myopia.

The primary visual cortex (V1) receives input from the dLGN and the SC (121). Additionally, V1 plays a crucial role in spatial frequency selectivity, with studies indicating that varying spatial frequencies can influence the development of myopia (122). Moreover, V1 is involved in the processing of ocular dominance and has been shown to play a role in the formation of amblyopia. Zhao’s study demonstrated that in guinea pigs with concave lens-induced myopia, the levels of GABA and the mRNA of GABA receptors in the visual cortex were elevated (123). Nakadate’s study discovered that monocular deprivation during the critical period of ocular dominance plasticity markedly altered both the quantity and pattern of c-Fos expression in the visual cortex. The most sensitive indicator was the number of c-Fos positive cells in layer IV of the binocular subfields of the primary visual cortex (Oc1B) ipsilateral to the stimulated eye (124). A reduction in the plasticity of neurons in the visual cortex can lead to deficits in contrast sensitivity, stereopsis, eye movement, and visual attention capabilities (125). Current research indicates that contrast sensitivity plays a role in the development of myopia (126). Figure 1 illustrates intraocular structures and potential central pathways involved in myopia.

In conclusion, current research indicates that central mechanisms play a significant role in the development and progression of myopia. Historically, the limitations of available methods have hindered our understanding of these central mechanisms. However, recent advancements in exploring neural circuits provide an opportunity to identify specific brain nuclei and neuron types involved in myopia. Additionally, investigating the potential interactions between central and intraocular mechanisms could offer deeper insights into the complex pathophysiology of myopia. Future studies should focus on utilizing these advanced techniques to unravel the precise contributions of central factors and their interplay with intraocular processes, it is hoped that artificial creation of optical defocus cues could be applied in humans, with the signals transmitted from the retinal ganglion cells to the brain to help control myopia progression. Such approaches would be non-invasive, safe, and convenient. It has been demonstrated that artificially altering defocus images can compensate for changes in eye growth (127–130). Additionally, besides defocus cues, other visual signals, such as contrast sensitivity (126, 131) and spatial frequency (132, 133), could also potentially play a role.

## References

[1] Morgan IG Ohno-Matsui K Saw SM. Myopia. Lancet. (2012) 379:1739–48. doi: 10.1016/s0140-6736(12)60272-4 22559900

[2] Holden BA Fricke TR Wilson DA Jong M Naidoo KS Sankaridurg P. Global prevalence of myopia and high myopia and temporal trends from 2000 through 2050. Ophthalmology. (2016) 123:1036–42. doi: 10.1016/j.ophtha.2016.01.006, PMID: 26875007

[3] Baird PN Saw SM Lanca C Guggenheim JA Smith Iii EL Zhou X. Myopia. Nat Rev Dis Primers. (2020) 6:99. doi: 10.1038/s41572-020-00231-4 33328468

[4] GBD 2019 Blindness and Vision Impairment Collaborators. Trends in prevalence of blindness and distance and near vision impairment over 30 years: an analysis for the global burden of disease study. Health. (2021) 9:e130–43. doi: 10.1016/s2214-109x(20)30425-3, PMID: 33275950 PMC7820390

[5] Ohno-Matsui K Lai TY Lai CC Cheung CMG. Updates of pathologic myopia. Prog Retin Eye Res. (2016) 52:156–87. doi: 10.1016/j.preteyeres.2015.12.001 26769165

[6] Wildsoet CF Chia A Cho P Guggenheim JA Polling JR Read S. IMI - interventions myopia institute: interventions for controlling myopia onset and progression report. Invest Ophthalmol Vis Sci. (2019) 60:M106–m131. doi: 10.1167/iovs.18-25958, PMID: 30817829

[7] Agyekum S Chan PP Adjei PE Zhang Y Huo Z Yip BHK. Cost-effectiveness analysis of myopia progression interventions in children. JAMA Netw Open. (2023) 6:e2340986. doi: 10.1001/jamanetworkopen.2023.40986, PMID: 37917061 PMC10623196

[8] Norton TT Essinger JA McBrien NA. Lid-suture myopia in tree shrews with retinal ganglion cell blockade. Vis Neurosci. (1994) 11:143–53. doi: 10.1017/s0952523800011184, PMID: 8011577

[9] McFadden SA Wildsoet C. The effect of optic nerve section on form deprivation myopia in the guinea pig. J Comp Neurol. (2020) 528:2874–87. doi: 10.1002/cne.24961, PMID: 32484917 PMC7541599

[10] Gong X Wu XH Liu AL Qian KW Li YY Ma YY. Optic nerve crush modulates refractive development of the C57BL/6 mouse by changing multiple ocular dimensions. Brain Res. (2020) 1726:146537. doi: 10.1016/j.brainres.2019.146537, PMID: 31672473

[11] Tkatchenko TV Troilo D Benavente-Perez A Tkatchenko AV. Gene expression in response to optical defocus of opposite signs reveals bidirectional mechanism of visually guided eye growth. PLoS Biol. (2018) 16:e2006021. doi: 10.1371/journal.pbio.2006021, PMID: 30300342 PMC6177118

[12] Wallman J Winawer J. Homeostasis of eye growth and the question of myopia. Neuron. (2004) 43:447–68. doi: 10.1016/j.neuron.2004.08.008, PMID: 15312645

[13] Flitcroft DI. Is myopia a failure of homeostasis? Exp Eye Res. (2013) 114:16–24. doi: 10.1016/j.exer.2013.02.008 23454097

[14] Benavente-Perez A Nour A Troilo D. Axial eye growth and refractive error development can be modified by exposing the peripheral retina to relative myopic or hyperopic defocus. Invest Ophthalmol Vis Sci. (2014) 55:6765–73. doi: 10.1167/iovs.14-14524, PMID: 25190657 PMC4209715

[15] Dowling JE Ehinger B. The interplexiform cell system. I. Synapses of the dopaminergic neurons of the goldfish retina. Proc R Soc Lond B Biol Sci. (1978) 201:7–26. doi: 10.1098/rspb.1978.0030 27792

[16] Nguyen-Legros J Versaux-Botteri C Savy C. Dopaminergic and GABAergic retinal cell populations in mammals. Microsc Res Tech. (1997) 36:26–42. doi: 10.1002/(sici)1097-0029(19970101)36:1<26::Aid-jemt3>3.0.Co;2-x, PMID: 9031259

[17] Dumitrescu ON Pucci FG Wong KY Berson DM. Ectopic retinal ON bipolar cell synapses in the OFF inner plexiform layer: contacts with dopaminergic amacrine cells and melanopsin ganglion cells. J Comp Neurol. (2009) 517:226–44. doi: 10.1002/cne.22158, PMID: 19731338 PMC3296562

[18] Zhao X Wong KY Zhang DQ. Mapping physiological inputs from multiple photoreceptor systems to dopaminergic amacrine cells in the mouse retina. Sci Rep. (2017) 7:7920. doi: 10.1038/s41598-017-08172-x, PMID: 28801634 PMC5554153

[19] Farshi P Fyk-Kolodziej B Krolewski DM Walker PD Ichinose T. Dopamine D1 receptor expression is bipolar cell type-specific in the mouse retina. J Comp Neurol. (2016) 524:2059–79. doi: 10.1002/cne.23932, PMID: 26587737 PMC4860096

[20] Chalupa LM Günhan E. Development of on and off retinal pathways and retinogeniculate projections. Prog Retin Eye Res. (2004) 23:31–51. doi: 10.1016/j.preteyeres.2003.10.001, PMID: 14766316

[21] Travis AM Heflin SJ Hirano AA Brecha NC Arshavsky VY. Dopamine-dependent sensitization of rod bipolar cells by GABA is conveyed through wide-field Amacrine cells. J Neurosci. (2018) 38:723–32. doi: 10.1523/jneurosci.1994-17.2017, PMID: 29217689 PMC5777116

[22] Ling Y Wang Y Ye J Luan C Bi A Gu Y. Changes in intrinsically photosensitive retinal ganglion cells, dopaminergic Amacrine cells, and their connectivity in the retinas of lid suture myopia. Invest Ophthalmol Vis Sci. (2024) 65:8. doi: 10.1167/iovs.65.11.8, PMID: 39230992 PMC11379095

[23] Beaulieu JM Gainetdinov RR. The physiology, signaling, and pharmacology of dopamine receptors. Pharmacol Rev. (2011) 63:182–217. doi: 10.1124/pr.110.002642 21303898

[24] Zhou X Pardue MT Iuvone PM Qu J. Dopamine signaling and myopia development: what are the key challenges. Prog Retin Eye Res. (2017) 61:60–71. doi: 10.1016/j.preteyeres.2017.06.003, PMID: 28602573 PMC5653403

[25] Shu Z Chen K Wang Q Wu H Zhu Y Tian R. The role of retinal dopamine D1 receptors in ocular growth and myopia development in mice. J Neurosci. (2023) 43:8231–42. doi: 10.1523/jneurosci.1196-23.2023, PMID: 37751999 PMC10697406

[26] Feldkaemper M Schaeffel F. An updated view on the role of dopamine in myopia. Exp Eye Res. (2013) 114:106–19. doi: 10.1016/j.exer.2013.02.007, PMID: 23434455

[27] Huang F Shu Z Huang Q Chen K Yan W Wu W. Retinal dopamine D2 receptors participate in the development of myopia in mice. Invest Ophthalmol Vis Sci. (2022) 63:24. doi: 10.1167/iovs.63.1.24, PMID: 35050306 PMC8787610

[28] Huang F Zhang L Wang Q Yang Y Li Q Wu Y. Dopamine D1 receptors contribute critically to the Apomorphine-induced inhibition of form-deprivation myopia in mice. Invest Ophthalmol Vis Sci. (2018) 59:2623–34. doi: 10.1167/iovs.17-22578 29847669

[29] Huang F Wang Q Yan T Tang J Hou X Shu Z. The role of the dopamine D2 receptor in form-deprivation myopia in mice: studies with full and partial D2 receptor agonists and knockouts. Invest Ophthalmol Vis Sci. (2020) 61:47. doi: 10.1167/iovs.61.6.47, PMID: 32572456 PMC7415310

[30] Chakraborty R Yang V Park HN Landis EG Dhakal S Motz CT. Lack of cone mediated retinal function increases susceptibility to form-deprivation myopia in mice. Exp Eye Res. (2019) 180:226–30. doi: 10.1016/j.exer.2018.12.021, PMID: 30605665 PMC6642639

[31] Taylor CP Shepard TG Rucker FJ Eskew RT Jr. Sensitivity to S-cone stimuli and the development of myopia. Invest Ophthalmol Vis Sci. (2018) 59:4622–30. doi: 10.1167/iovs.18-24113, PMID: 30242363 PMC6138264

[32] Fu Q Zhang Y Chen L Dong M Tang W Chen S. Near work induces myopia in Guinea pigs. Exp Eye Res. (2022) 224:109202. doi: 10.1016/j.exer.2022.109202, PMID: 35961425

[33] Gisbert S Schaeffel F. M to L cone ratios determine eye sizes and baseline refractions in chickens. Exp Eye Res. (2018) 172:104–11. doi: 10.1016/j.exer.2018.03.029, PMID: 29608907 PMC6013296

[34] Liang H Crewther DP Gillard Crewther S Barila AM. A role for photoreceptor outer segments in the induction of deprivation myopia. Vis Res. (1995) 35:1217–25. doi: 10.1016/0042-6989(94)00241-d, PMID: 7610583

[35] Smith EL 3rd Hung LF Huang J. Relative peripheral hyperopic defocus alters central refractive development in infant monkeys. Vis Res. (2009) 49:2386–92. doi: 10.1016/j.visres.2009.07.011, PMID: 19632261 PMC2745495

[36] Warwick RA Heukamp AS Riccitelli S Rivlin-Etzion M. Dopamine differentially affects retinal circuits to shape the retinal code. J Physiol. (2023) 601:1265–86. doi: 10.1113/jp284215, PMID: 36807203

[37] Nomi Y Iwasaki-Kurashige K Matsumoto H. Therapeutic effects of anthocyanins for vision and eye health. Molecules. (2019) 24:24. doi: 10.3390/molecules24183311, PMID: 31514422 PMC6767261

[38] Pérez-Fernández V Milosavljevic N Allen AE Vessey KA Jobling AI Fletcher EL. Rod photoreceptor activation alone defines the release of dopamine in the retina. Curr Biol. (2019) 29:763–774.e5. doi: 10.1016/j.cub.2019.01.042, PMID: 30799247

[39] Chakraborty R Park H Aung MH Tan CC Sidhu CS Iuvone PM. Comparison of refractive development and retinal dopamine in OFF pathway mutant and C57BL/6J wild-type mice. Mol Vis. (2014) 20:1318–27. PMID: 25352740 PMC4169773

[40] Paylakhi S Labelle-Dumais C Tolman NG Sellarole MA Seymens Y Saunders J. Müller glia-derived PRSS56 is required to sustain ocular axial growth and prevent refractive error. PLoS Genet. (2018) 14:e1007244. doi: 10.1371/journal.pgen.1007244, PMID: 29529029 PMC5864079

[41] Vecino E Rodriguez FD Ruzafa N Pereiro X Sharma SC. Glia-neuron interactions in the mammalian retina. Prog Retin Eye Res. (2016) 51:1–40. doi: 10.1016/j.preteyeres.2015.06.003, PMID: 26113209

[42] Zhang X Yu X Wen Y Jin L Zhang L Zhu H. Functions of retinal astrocytes and Müller cells in mammalian myopia. BMC Ophthalmol. (2022) 22:451. doi: 10.1186/s12886-022-02643-0, PMID: 36418970 PMC9686084

[43] Lange J Yafai Y Reichenbach A Wiedemann P Eichler W. Regulation of pigment epithelium-derived factor production and release by retinal glial (Müller) cells under hypoxia. Invest Ophthalmol Vis Sci. (2008) 49:5161–7. doi: 10.1167/iovs.08-2201, PMID: 18676622

[44] Barnard AR Hattar S Hankins MW Lucas RJ. Melanopsin regulates visual processing in the mouse retina. Curr Biol. (2006) 16:389–95. doi: 10.1016/j.cub.2005.12.045, PMID: 16488873

[45] Liu AL Liu YF Wang G Shao YQ Yu CX Yang Z. The role of ipRGCs in ocular growth and myopia development. Sci Adv. (2022) 8:eabm9027. doi: 10.1126/sciadv.abm9027, PMID: 35675393 PMC9176740

[46] Wu XH Li YY Zhang PP Qian KW Ding JH Hu G. Unaltered retinal dopamine levels in a C57BL/6 mouse model of form-deprivation myopia. Invest Ophthalmol Vis Sci. (2015) 56:967–77. doi: 10.1167/iovs.13-13362 25604682

[47] Goetz J Jessen ZF Jacobi A Mani A Cooler S Greer D. Unified classification of mouse retinal ganglion cells using function, morphology, and gene expression. Cell Rep. (2022) 40:111040. doi: 10.1016/j.celrep.2022.111040, PMID: 35830791 PMC9364428

[48] Pan F. Defocused image changes signaling of ganglion cells in the mouse retina. Cells. (2019) 8:8. doi: 10.3390/cells8070640, PMID: 31247948 PMC6678497

[49] Wang Q So C Zuo B Banerjee S Qiu C Ting Z. Retinal ganglion cells encode differently in the myopic mouse retina? Exp Eye Res. (2023) 234:109616. doi: 10.1016/j.exer.2023.109616 37580002

[50] Gallego-Ortega A Norte-Muñoz M di Pierdomenico J Avilés-Trigueros M de la Villa P Valiente-Soriano FJ. Alpha retinal ganglion cells in pigmented mice retina: number and distribution. Front Neuroanat. (2022) 16:1054849. doi: 10.3389/fnana.2022.1054849, PMID: 36530520 PMC9751430

[51] Krieger B Qiao M Rousso DL Sanes JR Meister M. Four alpha ganglion cell types in mouse retina: function, structure, and molecular signatures. PLoS One. (2017) 12:e0180091. doi: 10.1371/journal.pone.0180091, PMID: 28753612 PMC5533432

[52] Jiang X Pardue MT Mori K Ikeda SI Torii H D’Souza S. Violet light suppresses lens-induced myopia via neuropsin (OPN5) in mice. Proc Natl Acad Sci USA. (2021) 118:118. doi: 10.1073/pnas.2018840118, PMID: 34031241 PMC8179197

[53] Mani A Schwartz GW. Circuit mechanisms of a retinal ganglion cell with stimulus-dependent response latency and activation beyond its dendrites. Curr Biol. (2017) 27:471–82. doi: 10.1016/j.cub.2016.12.033, PMID: 28132812 PMC5319888

[54] Zhang Y Liu Y Wildsoet CF. Bidirectional, optical sign-dependent regulation of BMP2 gene expression in chick retinal pigment epithelium. Invest Ophthalmol Vis Sci. (2012) 53:6072–80. doi: 10.1167/iovs.12-9917, PMID: 22879416 PMC4113186

[55] Troilo D Smith EL Nickla DL Ashby R Tkatchenko AV Ostrin LA. IMI - report on experimental models of Emmetropization and myopia. Invest Ophthalmol Vis Sci. (2019) 60:M31–m88. doi: 10.1167/iovs.18-25967, PMID: 30817827 PMC6738517

[56] Liang H Crewther SG Crewther DP Junghans BM. Structural and elemental evidence for edema in the retina, retinal pigment epithelium, and choroid during recovery from experimentally induced myopia. Invest Ophthalmol Vis Sci. (2004) 45:2463–74. doi: 10.1167/iovs.03-1009, PMID: 15277465

[57] Nickla DL Wallman J. The multifunctional choroid. Prog Retin Eye Res. (2010) 29:144–68. doi: 10.1016/j.preteyeres.2009.12.002, PMID: 20044062 PMC2913695

[58] Zhou X Zhang S Zhang G Chen Y Lei Y Xiang J. Increased choroidal blood perfusion can inhibit form deprivation myopia in Guinea pigs. Invest Ophthalmol Vis Sci. (2020) 61:25. doi: 10.1167/iovs.61.13.25 PMC7683853 33211066

[59] Zhou X Zhang S Yang F Yang Y Huang Q Huang C. Decreased choroidal blood perfusion induces myopia in Guinea pigs. Invest Ophthalmol Vis Sci. (2021) 62:30. doi: 10.1167/iovs.62.15.30, PMID: 34967855 PMC8740532

[60] Gawne TJ Siegwart JT Ward AH Norton TT. The wavelength composition and temporal modulation of ambient lighting strongly affect refractive development in young tree shrews. Exp Eye Res. (2017) 155:75–84. doi: 10.1016/j.exer.2016.12.004, PMID: 27979713 PMC5359068

[61] Ohlendorf A Schaeffel F. Contrast adaptation induced by defocus - a possible error signal for emmetropization? Vis Res. (2009) 49:249–56. doi: 10.1016/j.visres.2008.10.016, PMID: 19000917

[62] Wu H Chen W Zhao F Zhou Q Reinach PS Deng L. Scleral hypoxia is a target for myopia control. Proc Natl Acad Sci USA. (2018) 115:E7091–e7100. doi: 10.1073/pnas.1721443115, PMID: 29987045 PMC6064999

[63] Troilo D Gottlieb MD Wallman J. Visual deprivation causes myopia in chicks with optic nerve section. Curr Eye Res. (1987) 6:993–9. doi: 10.3109/02713688709034870, PMID: 3665562

[64] Diether S Schaeffel F. Local changes in eye growth induced by imposed local refractive error despite active accommodation. Vis Res. (1997) 37:659–68. doi: 10.1016/s0042-6989(96)00224-6, PMID: 9156210

[65] Zhao F Zhang D Zhou Q Zhao F He M Yang Z. Scleral HIF-1α is a prominent regulatory candidate for genetic and environmental interactions in human myopia pathogenesis. EBioMedicine. (2020) 57:102878. doi: 10.1016/j.ebiom.2020.102878, PMID: 32652319 PMC7348000

[66] Kang MT Wang B Ran AR Gan J du J Yusufu M. Brain activation induced by myopic and hyperopic defocus from spectacles. Front Hum Neurosci. (2021) 15:711713. doi: 10.3389/fnhum.2021.711713, PMID: 34594194 PMC8477670

[67] Zhang XW Dai RP Cheng GW Zhang WH Long Q. Altered amplitude of low-frequency fluctuations and default mode network connectivity in high myopia: a resting-state fMRI study. Int J Ophthalmol. (2020) 13:1629–36. doi: 10.18240/ijo.2020.10.18, PMID: 33078115 PMC7511385

[68] Ji Y Cheng Q Fu WW Zhong PP Huang SQ Chen XL. Exploration of abnormal dynamic spontaneous brain activity in patients with high myopia via dynamic regional homogeneity analysis. Front Hum Neurosci. (2022) 16:959523. doi: 10.3389/fnhum.2022.959523, PMID: 35992950 PMC9390771

[69] Liu CH Li F Li SF Wang YJ Tie CL Wu HY. Abnormal baseline brain activity in bipolar depression: a resting state functional magnetic resonance imaging study. Psychiatry Res. (2012) 203:175–9. doi: 10.1016/j.pscychresns.2012.02.007, PMID: 23017873

[70] Shao Y Huang X Cai F Hu PH Zhong Y Zhang Y. Disturbed spontaneous brain-activity pattern in patients with optic neuritis using amplitude of low-frequency fluctuation: a functional magnetic resonance imaging study. Neuropsychiatr Dis Treat. (2015) 11:3075–83. doi: 10.2147/ndt.S92497, PMID: 26719692 PMC4689287

[71] Li T Liu Z Li J Liu Z Tang Z Xie X. Altered amplitude of low-frequency fluctuation in primary open-angle glaucoma: a resting-state FMRI study. Invest Ophthalmol Vis Sci. (2014) 56:322–9. doi: 10.1167/iovs.14-14974, PMID: 25525176

[72] Min YL Su T Shu YQ Liu WF Chen LL Shi WQ. Altered spontaneous brain activity patterns in strabismus with amblyopia patients using amplitude of low-frequency fluctuation: a resting-state fMRI study. Neuropsychiatr Dis Treat. (2018) 14:2351–9. doi: 10.2147/ndt.S171462, PMID: 30275692 PMC6157537

[73] Zhang X Liu L Jin X Han S Yang F Xu Y. Altered time-varying local spontaneous brain activity pattern in patients with high myopia: a dynamic amplitude of low-frequency fluctuations study. Neuroradiology. (2023) 65:157–66. doi: 10.1007/s00234-022-03033-5, PMID: 35953566

[74] Yu-Feng Z Yong H Chao-Zhe Z Qing-Jiu C Man-Qiu S Meng L. Altered baseline brain activity in children with ADHD revealed by resting-state functional MRI. Brain and Development. (2007) 29:83–91. doi: 10.1016/j.braindev.2006.07.002, PMID: 16919409

[75] Huang X Zhou FQ Hu YX Xu XX Zhou X Zhong YL. Altered spontaneous brain activity pattern in patients with high myopia using amplitude of low-frequency fluctuation: a resting-state fMRI study. Neuropsychiatr Dis Treat. (2016) 12:2949–56. doi: 10.2147/ndt.S118326, PMID: 27881920 PMC5115684

[76] Zhai L Li Q Wang T Dong H Peng Y Guo M. Altered functional connectivity density in high myopia. Behav Brain Res. (2016) 303:85–92. doi: 10.1016/j.bbr.2016.01.046 26808608

[77] Hu YX He JR Yang B Huang X Li YP Zhou FQ. Abnormal resting-state functional network centrality in patients with high myopia: evidence from a voxel-wise degree centrality analysis. Int J Ophthalmol. (2018) 11:1814–20. doi: 10.18240/ijo.2018.11.13, PMID: 30450313 PMC6232325

[78] Ji Y Shi L Cheng Q Fu WW Zhong PP Huang SQ. Abnormal large-scale neuronal network in high myopia. Front Hum Neurosci. (2022) 16:870350. doi: 10.3389/fnhum.2022.870350, PMID: 35496062 PMC9051506

[79] Dronkers NF Wilkins DP van Valin RD Jr Redfern BB Jaeger JJ. Lesion analysis of the brain areas involved in language comprehension. Cognition. (2004) 92:145–77. doi: 10.1016/j.cognition.2003.11.002, PMID: 15037129

[80] Dal Monte O Schintu S Pardini M Berti A Wassermann EM Grafman J. The left inferior frontal gyrus is crucial for reading the mind in the eyes: brain lesion evidence. Cortex. (2014) 58:9–17. doi: 10.1016/j.cortex.2014.05.002 24946302

[81] Hampshire A Chamberlain SR Monti MM Duncan J Owen AM. The role of the right inferior frontal gyrus: inhibition and attentional control. NeuroImage. (2010) 50:1313–9. doi: 10.1016/j.neuroimage.2009.12.109, PMID: 20056157 PMC2845804

[82] Vicente AF Bermudez MA Romero MC Perez R Gonzalez F. Putamen neurons process both sensory and motor information during a complex task. Brain Res. (2012) 1466:70–81. doi: 10.1016/j.brainres.2012.05.037, PMID: 22640776

[83] Dunkley BT Freeman TC Muthukumaraswamy SD Singh KD. Cortical oscillatory changes in human middle temporal cortex underlying smooth pursuit eye movements. Hum Brain Mapp. (2013) 34:837–51. doi: 10.1002/hbm.21478, PMID: 22110021 PMC6869956

[84] Huster RJ Enriquez-Geppert S Pantev C Bruchmann M. Variations in midcingulate morphology are related to ERP indices of cognitive control. Brain Struct Funct. (2014) 219:49–60. doi: 10.1007/s00429-012-0483-5, PMID: 23179865

[85] Megreli J Barak A Bez M Bez D Levine H. Association of Myopia with cognitive function among one million adolescents. BMC Public Health. (2020) 20:647. doi: 10.1186/s12889-020-08765-8, PMID: 32384882 PMC7206693

[86] Li Q Guo M Dong H Zhang Y Fu Y Yin X. Voxel-based analysis of regional gray and white matter concentration in high myopia. Vis Res. (2012) 58:45–50. doi: 10.1016/j.visres.2012.02.005, PMID: 22402232

[87] Wu YJ Wu N Huang X Rao J Yan L Shi L. Evidence of cortical thickness reduction and disconnection in high myopia. Sci Rep. (2020) 10:16239. doi: 10.1038/s41598-020-73415-3 33004887 PMC7530748

[88] Mirzajani A Sarlaki E Kharazi HH Tavan M. Effect of lens-induced myopia on visual cortex activity: a functional MR imaging study. AJNR Am J Neuroradiol. (2011) 32:1426–9. doi: 10.3174/ajnr.A2551, PMID: 21816915 PMC7964350

[89] Mirzajani A Ghorbani M Rasuli B Mahmoud-Pashazadeh A. Effect of induced high myopia on functional MRI signal changes. Phys Med. (2017) 37:32–6. doi: 10.1016/j.ejmp.2017.04.004, PMID: 28535912

[90] Nelles G Pscherer A de Greiff A Esser J. Brain activation of eye movements in subjects with refractive error. Brain. (2010) 2:57–62. doi: 10.2147/eb.s9823, PMID: 28539763 PMC5436169

[91] Cheng Y Huang X Hu YX Huang MH Yang B Zhou FQ. Comparison of intrinsic brain activity in individuals with low/moderate myopia versus high myopia revealed by the amplitude of low-frequency fluctuations. Acta Radiol. (2020) 61:496–507. doi: 10.1177/0284185119867633 31398992

[92] Raviola E Wiesel TN. An animal model of myopia. N Engl J Med. (1985) 312:1609–15. doi: 10.1056/nejm198506203122505 4000200

[93] Choh V Lew MY Nadel MW Wildsoet CF. Effects of interchanging hyperopic defocus and form deprivation stimuli in normal and optic nerve-sectioned chicks. Vis Res. (2006) 46:1070–9. doi: 10.1016/j.visres.2005.08.020, PMID: 16212999

[94] Wildsoet C. Neural pathways subserving negative lens-induced emmetropization in chicks--insights from selective lesions of the optic nerve and ciliary nerve. Curr Eye Res. (2003) 27:371–85. doi: 10.1076/ceyr.27.6.371.18188, PMID: 14704921

[95] Dillingham CM Guggenheim JA Erichsen JT. Disruption of the centrifugal visual system inhibits early eye growth in chicks. Invest Ophthalmol Vis Sci. (2013) 54:3632–43. doi: 10.1167/iovs.12-11548, PMID: 23599339

[96] Mcbrien NA Moghaddam HO Cottriall CL Leech EM Cornell LM. The effects of blockade of retinal cell action potentials on ocular growth, emmetropization and form deprivation myopia in young chicks. Vis Res. (1995) 35:1141–52. doi: 10.1016/0042-6989(94)00237-g, PMID: 7610575

[97] Choh V Padmanabhan V Li WS Sullivan AB Wildsoet CF. Colchicine attenuates compensation to negative but not to positive lenses in young chicks. Exp Eye Res. (2008) 86:260–70. doi: 10.1016/j.exer.2007.10.017, PMID: 18078935 PMC2440497

[98] Fischer AJ Morgan IG Stell WK. Colchicine causes excessive ocular growth and myopia in chicks. Vis Res. (1999) 39:685–97. doi: 10.1016/s0042-6989(98)00178-3, PMID: 10341956

[99] May CA Lütjen-Drecoll E. Morphology of the murine optic nerve. Invest Ophthalmol Vis Sci. (2002) 43:2206–12. PMID: 12091418

[100] Mojumder DK Frishman LJ Otteson DC Sherry DM. Voltage-gated sodium channel alpha-subunits Na(v)1.1, Na(v)1.2, and Na(v)1.6 in the distal mammalian retina. Mol Vis. (2007) 13:2163–82. PMID: 18079688

[101] Labandeira-Garcia JL Guerra-Seijas MJ Gonzalez F Perez R Acuña C. Location of neurons projecting to the retina in mammals. Neurosci Res. (1990) 8:291–302. doi: 10.1016/0168-0102(90)90035-d, PMID: 2175862

[102] Gastinger MJ Barber AJ Khin SA McRill C Gardner TW Marshak DW. Abnormal centrifugal axons in streptozotocin-diabetic rat retinas. Invest Ophthalmol Vis Sci. (2001) 42:2679–85. PMID: 11581216 PMC3341734

[103] Esposti F Johnston J Rosa JM Leung KM Lagnado L. Olfactory stimulation selectively modulates the OFF pathway in the retina of zebrafish. Neuron. (2013) 79:97–110. doi: 10.1016/j.neuron.2013.05.001, PMID: 23849198 PMC3710973

[104] Chen WY Han X Cui LJ Yu CX Sheng WL Yu J. Cell-subtype-specific remodeling of intrinsically photosensitive retinal ganglion cells in Streptozotocin-induced diabetic mice. Diabetes. (2021) 70:1157–69. doi: 10.2337/db20-0775, PMID: 33574020

[105] Sikka G Hussmann GP Pandey D Cao S Hori D Park JT. Melanopsin mediates light-dependent relaxation in blood vessels. Proc Natl Acad Sci U S A. (2014) 111:17977–82. doi: 10.1073/pnas.1420258111, PMID: 25404319 PMC4273372

[106] Benavidez NL Bienkowski MS Zhu M Garcia LH Fayzullina M Gao L. Organization of the inputs and outputs of the mouse superior colliculus. Nat Commun. (2021) 12:4004. doi: 10.1038/s41467-021-24241-2, PMID: 34183678 PMC8239028

[107] Seabrook TA Burbridge TJ Crair MC Huberman AD. Architecture, function, and assembly of the mouse visual system. Annu Rev Neurosci. (2017) 40:499–538. doi: 10.1146/annurev-neuro-071714-033842, PMID: 28772103

[108] Ellis EM Gauvain G Sivyer B Murphy GJ. Shared and distinct retinal input to the mouse superior colliculus and dorsal lateral geniculate nucleus. J Neurophysiol. (2016) 116:602–10. doi: 10.1152/jn.00227.2016, PMID: 27169509 PMC4982907

[109] Furigo IC de Oliveira WF de Oliveira AR Comoli E Baldo MV Mota-Ortiz SR. The role of the superior colliculus in predatory hunting. Neuroscience. (2010) 165:1–15. doi: 10.1016/j.neuroscience.2009.10.004, PMID: 19825395

[110] Yilmaz M Meister M. Rapid innate defensive responses of mice to looming visual stimuli. Curr Biol. (2013) 23:2011–5. doi: 10.1016/j.cub.2013.08.015, PMID: 24120636 PMC3809337

[111] Lee JM Hill RM. Responses of midbrain cells to blur. Pflugers Arch. (1972) 336:213–6. doi: 10.1007/bf00590045, PMID: 4673501

[112] Gehr C Sibille J Kremkow J. Retinal input integration in excitatory and inhibitory neurons in the mouse superior colliculus in vivo. eLife. (2023) 12:12. doi: 10.7554/eLife.88289, PMID: 37682267 PMC10491433

[113] Li YT Meister M. Functional cell types in the mouse superior colliculus. eLife. (2023) 12:12. doi: 10.7554/eLife.82367, PMID: 37073860 PMC10121220

[114] Wiesel TN Hubel DH. Effects of visual deprivation on morphology and physiology of cells in the CATS lateral geniculate body. J Neurophysiol. (1963) 26:978–93. doi: 10.1152/jn.1963.26.6.978 14084170

[115] Nickla DL. Ocular diurnal rhythms and eye growth regulation: where we are 50 years after Lauber. Exp Eye Res. (2013) 114:25–34. doi: 10.1016/j.exer.2012.12.013, PMID: 23298452 PMC3742730

[116] Burfield HJ Patel NB Ostrin LA. Ocular biometric diurnal rhythms in Emmetropic and myopic adults. Invest Ophthalmol Vis Sci. (2018) 59:5176–87. doi: 10.1167/iovs.18-25389, PMID: 30372744 PMC6203176

[117] Li JY Schmidt TM. Divergent projection patterns of M1 ipRGC subtypes. J Comp Neurol. (2018) 526:2010–8. doi: 10.1002/cne.24469, PMID: 29888785 PMC6158116

[118] Baver SB Pickard GE Sollars PJ Pickard GE. Two types of melanopsin retinal ganglion cell differentially innervate the hypothalamic suprachiasmatic nucleus and the olivary pretectal nucleus. Eur J Neurosci. (2008) 27:1763–70. doi: 10.1111/j.1460-9568.2008.06149.x, PMID: 18371076

[119] Li L Yu Y Zhuang Z Wu Q Lin S Hu J. Circadian rhythm, ipRGCs, and dopamine signalling in myopia. Graefes Arch Clin Exp Ophthalmol. (2023) 262:983–90. doi: 10.1007/s00417-023-06276-x, PMID: 37864638

[120] Ralph MR Foster RG Davis FC Menaker M. Transplanted suprachiasmatic nucleus determines circadian period. Science. (1990) 247:975–8. doi: 10.1126/science.2305266 2305266

[121] Glickfeld LL Olsen SR. Higher-order areas of the mouse visual cortex. Annu Rev Vis Sci. (2017) 3:251–73. doi: 10.1146/annurev-vision-102016-061331 28746815

[122] Zhi Z Pan M Xie R Xiong S Zhou X Qu J. The effect of temporal and spatial stimuli on the refractive status of guinea pigs following natural emmetropization. Invest Ophthalmol Vis Sci. (2013) 54:890–7. doi: 10.1167/iovs.11-8064, PMID: 23307951

[123] Zhao W Bi AL Xu CL Ye X Chen MQ Wang XT. GABA and GABA receptors alterations in the primary visual cortex of concave lens-induced myopic model. Brain Res Bull. (2017) 130:173–9. doi: 10.1016/j.brainresbull.2017.01.017, PMID: 28163071

[124] Nakadate K Imamura K Watanabe Y. Effects of monocular deprivation on the spatial pattern of visually induced expression of c-Fos protein. Neuroscience. (2012) 202:17–28. doi: 10.1016/j.neuroscience.2011.12.004, PMID: 22178607

[125] Yu X Zhao F Li X Lu W Zhao L Li D. Resting-state functional connectivity of the primary visual cortex in children with Anisometropia amblyopia. Ophthalmic Res. (2024) 67:1–281. doi: 10.1159/000538380, PMID: 38588644

[126] Poudel S Jin J Rahimi-Nasrabadi H Dellostritto S Dul MW Viswanathan S. Contrast sensitivity of ON and OFF human retinal pathways in myopia. J Neurosci. (2024) 44:e1487232023. doi: 10.1523/jneurosci.1487-23.2023 38050109 PMC10860621

[127] Jiang X Kurihara T Kunimi H Miyauchi M Ikeda SI Mori K. A highly efficient murine model of experimental myopia. Sci Rep. (2018) 8:2026. doi: 10.1038/s41598-018-20272-w, PMID: 29391484 PMC5794929

[128] Graham B Judge SJ. The effects of spectacle wear in infancy on eye growth and refractive error in the marmoset (*Callithrix jacchus*). Vis Res. (1999) 39:189–206. doi: 10.1016/s0042-6989(98)00189-8, PMID: 10326130

[129] Hung LF Crawford MLJ Smith EL. Spectacle lenses alter eye growth and the refractive status of young monkeys. Nat Med. (1995) 1:761–5. doi: 10.1038/nm0895-761, PMID: 7585177

[130] Smith EL 3rd. Spectacle lenses and emmetropization: the role of optical defocus in regulating ocular development. Optom Vis Sci. (1998) 75:388–98. doi: 10.1097/00006324-199806000-00023, PMID: 9661208

[131] Liou SW Chiu CJ. Myopia and contrast sensitivity function. Curr Eye Res. (2001) 22:81–4. doi: 10.1076/ceyr.22.2.81.5530 11402383

[132] Li DL Dong XX Yang JL Lanca C Grzybowski A Pan CW. Lower indoor spatial frequency increases the risk of myopia in children. Br J Ophthalmol. (2024):bjo-2024-325888. doi: 10.1136/bjo-2024-325888, PMID: 39122351

[133] Flitcroft DI Harb EN Wildsoet CF. The spatial frequency content of urban and indoor environments as a potential risk factor for myopia development. Invest Ophthalmol Vis Sci. (2020) 61:42. doi: 10.1167/iovs.61.11.42, PMID: 32986814 PMC7533745

